# Neosporosis in 21 adult dogs, 2010‐2023

**DOI:** 10.1111/jvim.17219

**Published:** 2024-10-23

**Authors:** Alexandra Kennedy, Joanna D. White, Georgina Child

**Affiliations:** ^1^ Small Animal Specialist Hospital Ryde New South Wales Australia

**Keywords:** creatinine kinase, hepatopathy, immunosuppression, meningoencephalitis, myopathy, outcome

## Abstract

**Background:**

Limited information is available regarding the clinical features, treatment, and prognosis of neosporosis in adult dogs.

**Objective:**

Describe the clinical signs, laboratory findings, magnetic resonance imaging (MRI) findings, treatment and outcome in adult dogs (>6 months) diagnosed with neosporosis based on consistent clinical signs and positive serology (titer ≥1 : 800) at a referral hospital in Sydney, Australia.

**Animals:**

Twenty‐one client‐owned dogs.

**Methods:**

Retrospective case series of affected dogs between 2010 and 2023. Survival times were determined from onset of clinical signs to date of death or censoring.

**Results:**

Clinical signs varied, and were indicative of generalized myopathy (6 dogs), multifocal intracranial disease (7 dogs), myelopathy (4 dogs), polyneuropathy (2 dogs) and single cases of focal myopathy and cerebellar disease. Serum creatine kinase activity was markedly increased (median, 3369 U/L) in most dogs. The most common MRI abnormalities were multifocal intracranial abnormalities (7/13 dogs) and muscle changes (5/13 dogs) whereas T2‐weighted cerebellar abnormalities (2/13 dogs) and cerebellar atrophy (1/13) were less common. Treatment response was complete (resolution to normal) in 8 dogs, incomplete (persistent neurological deficits) in 6 dogs, but there was minimal response in 7 dogs. Thirteen dogs (62%) were alive after 6 months and 12 dogs (57%) alive after 1 year. Relapse was common, with 4 dogs experiencing at least 1 relapse event during the follow‐up period.

**Conclusion and Clinical Importance:**

Adult‐onset neosporosis is uncommon and has variable clinical presentations. Treatment response also is variable, and relapse can occur, even among patients that respond completely to initial treatment.

**Abbreviations**
ALT: alanine aminotransferase
CK: creatine kinase
CKCS: Cavalier King Charles spaniel
CSF: cerebrospinal fluid
D1: dog one
DCM: dilated cardiomyopathy
IFAT: indirect fluorescence antibody test
IHC: immunohistochemistry
IMTP: immune mediated thrombocytopenia
MRI: magnetic resonance imaging
MUO: meningoencephalitis of unknown origin
PCR: polymerase chain reaction
WHWT: West Highland white terrier

## 1 INTRODUCTION

Neospora is an obligate intracellular protozoan with worldwide distribution that uncommonly causes disease in dogs, but has substantial impact on cattle production and fertility.^1^, ^2^ Dogs are the definitive host for this protozoan, which undergoes an entero‐epithelial life cycle with cattle as the typical intermediate host, although other species have been described.^2^ Vertical transmission via the transplacental route is reported to be the most common route of infection in dogs, leading to formation of bradyzoite cysts and clinical disease sporadically.^2^ Exposure is postulated to be more likely in mixed breed dogs, but clinical disease is more likely to develop in purebred dogs.^2^ Horizontal transmission may occur through ingestion of oocysts in feces or ingestion of infected tissue from intermediate hosts.^2^

Clinical neosporosis is more common in dogs <6 months of age, with onset of clinical signs commonly at 3 to 9 weeks and is well documented.^2^ In puppies, transplacental infection can cause myositis, radiculoneuritis, and myelitis predominantly affecting the pelvic limbs and development of pelvic limb extensor rigidity associated with muscle contracture.^2^ Clinical neosporosis in dogs >6 months of age is considered a distinct disease syndrome from juvenile neosporosis and is thought to be secondary to reactivation of latent chronic infection, rather than initial progressive infection.^2^, ^3^

Antemortem diagnosis of neosporosis remains a clinical challenge, with gold standard diagnosis using detection of antigen in tissues often not feasible because of difficulty and practicality of obtaining samples of commonly affected tissue (nervous system or muscular tissue). Immunoglobulin G antibodies typically progressively increase to high concentrations during active disease.^4^ A presumptive clinical diagnosis is commonly made based neurological or neuromuscular abnormalities and positive serology, with exclusion of other causes.^2^, ^3^

Adult‐onset clinical neosporosis remains less understood compared to juvenile neosporosis, and often is diagnosed post‐mortem. A recent literature review identified 56 published cases across 40 publications, with 1 subsequent case resulting in a total 57 cases total in the veterinary literature at the time of writing, including neurological and cutaneous presentations.^5^, ^6^ Information on prognosis and treatment protocols is limited, because the majority of these case reports document diagnosis rather than treatment and outcome.

Our objective was to describe the clinicopathological features, treatment, and outcome of neosporosis in adult (>6 months of age) dogs.

## 2 METHODS

### 2.1 Study design

The medical record database (Ezyvet) at a single referral hospital was searched for dogs that had neospora antibody titer testing, PCR or immunohistochemistry (IHC) performed using search of billing codes for this retrospective case series from 2010 to April 2023. Antibody titer testing was performed by the Department of Primary Industries Animal Health Laboratory using an indirect fluorescence antibody test (IFAT) as previously described.^2^ Medical records of dogs with titers ≥1 : 800 or positive PCR or IHC were reviewed by the primary author. Data retrieved included signalment, risk factors including rural location and raw meat exposure, clinicopathological and serological findings, imaging and cerebrospinal fluid (CSF) abnormalities, treatments, survival, and clinical relapse.

### 2.2 Cases

To be included dogs had to:

1. develop neurological or neuromuscular abnormalities at >6 months of age, as determined by a board‐certified neurologist
2. And have:
  - neospora IFAT serum titer ≥1 : 800 or
  - positive neospora IFAT serum titer <1 : 800 and detection of antigen on PCR or IHC.

Dogs were considered adult at >6 months of age according to young adult definition as the time of cessation of rapid growth (6‐9 months of age, varying with breed and size).^7^ This age previously has been described as the point of distinction between dogs with the juvenile form and adult dogs with suspected reactivation of infection.^5^ The serology cut‐off ≥1 : 800 was selected as the clinically used threshold for likely disease, because a previous seroprevalence study suggested a serum titer of 1 : 800 was more appropriate than a titer of 1 : 200 in supporting a diagnosis of clinical neosporosis, with a seroprevalence of only 1.3% of clinically normal dogs in Sydney having a titer of ≥1 : 800.^3^, ^8^, ^9^

Using the search criteria, dogs <6 months of age at time of onset of clinical signs, dogs with active concurrent disease and dogs with inadequate follow‐up were excluded.

All dogs underwent physical and neurological examination by a board‐certified neurologist. Eighteen of 21 had routine hematology and 20/21 had routine serum biochemistry performed. One dog with a high index of suspicion of neosporosis because of known disease in other family members underwent serological testing only because of client financial constraints.

Seven dogs with increased alanine aminotransferase (ALT) activity had further diagnostic tests for hepatic disease before investigation of neurological or myopathic disease. Hepatic function testing was performed in 3 dogs, including bile acid testing in 3 and ammonia tolerance testing in 1. In 4 dogs, abdominal ultrasonography was performed by a board‐certified radiologist and cytology of hepatic aspirates was performed by a board‐certified pathologist. Toxoplasma serum IFAT was performed in 19 dogs. One dog with signs of masticatory myopathy had 2 M antibody titer testing performed.

Thirteen dogs had magnetic resonance imaging (MRI) performed.^1^ All images were reviewed by a board‐certified radiologist. Magnetic resonance imaging of the head and brain was performed in 10 dogs; the cervical, thoracolumbar, and caudal spinal region were imaged in 3, 4, and 3 dogs, respectively (Table S3).

Cerebrospinal fluid was collected by cisternal or lumbar puncture by a board‐certified neurologist and submitted for analysis in 6 dogs. Polymerase chain reaction was performed on CSF in 4 dogs.

Treatment protocol varied based on clinician experience over the time period. Dogs initially were treated with clindamycin 15 mg/kg PO q12h for 8 weeks. The first dog to be diagnosed was treated for 8 weeks and had recurrence of clinical signs within days of discontinuing treatment and was treated for a further 8 weeks. This experience resulted in ongoing recommendation of a 12‐16 week treatment period. However, as experience grew, dogs additionally were additionally with trimethoprim‐sulfonamide at 15‐20 mg/kg PO q12h for 12 weeks. Additionally, several dogs were treated with a short tapering course of corticosteroids at anti‐inflammatory dosages (1 mg/kg/day initially).

Descriptive statistics were determined for age and laboratory test results. Survival time was defined as the time between diagnosis and death. Outcome was determined by review of medical records and client communications. Dogs alive as of April 3, 2023 (5) were censored as were dogs lost to follow‐up (3) at their last known follow‐up date. All censored dogs had at least 12 months follow‐up available. Kaplan‐Meier survival analysis was performed and median survival times calculated.

Specific ethical committee consent was not required for this retrospective study. Owner information and consent forms including permission for sharing de‐identified clinical information are part of standard permission forms that are signed before hospital admission.

## 3 RESULTS

A total of 682 dogs had neospora antibody titer testing performed over the time period of the study (2010‐2023), with 26 dogs have a titer ≥1 : 800. One dog had IHC performed, which was positive with a titer of 1 : 400. Polymerase chain reaction on CSF was performed in 66 dogs and did not identify any additional cases. The PCR was positive in 1 dog that had concurrent serology ≥1 : 800. Medical records of these 27 dogs were reviewed. Of these dogs, 26 presented with neurological or neuromuscular signs. One dog with cutaneous neosporosis confirmed with IHC on a skin biopsy sample had active concurrent disease (bacterial endocarditis) at the time of neosporosis diagnosis and was excluded from analysis. Dogs <6 months of age at time of onset of clinical signs (4) and dogs with inadequate follow‐up (1) were excluded. Five dogs that had received corticosteroids for another condition before developing clinical signs of neosporosis were included. Clinical signs of all dogs' prior conditions were resolved before onset of clinical signs of neosporosis. The remaining 16 dogs had no history or evidence of concurrent disease.

Twenty‐one (9 neutered female, 10 neutered male, 2 intact male) dogs met the inclusion criteria. All dogs lived in Sydney, Australia at time of diagnosis, and were treated at a single referral hospital. Median age of onset of clinical signs was 2.5 years (range, 8 months‐10 years). Seventeen dogs were identified as purebred whereas 4 were crossbred. Affected breeds included greyhound (4), West Highland white terrier, Cavalier King Charles spaniel, pug and French bulldog (2 dogs each) and individual dogs that were bullmastiff, Bernese mountain dog, Doberman, Labrador retriever and Hungarian vizsla.

One dog had known prior rural exposure. The 4 greyhounds were previous racing dogs and had incomplete history before private ownership. Seven dogs had known exposure to raw beef in their diet.

Vertical transmission of infection was suspected in 3 dogs. Two dogs were from the same litter (French bulldogs) with a third littermate having a positive titer of 1 : 100 without clinical signs (not included). No titer results from the dam or other littermates were available. The third dog (Doberman) with suspected vertical transmission was 12 months of age at diagnosis. This dog's dam had a positive IFAT titer of 1 : 200, with a puppy from a subsequent litter presenting with the typical juvenile neosporosis. No convincing evidence of vertical vs horizontal transmission was identified in any of the other cases.

Two affected French bulldogs developed clinical signs after receiving approximately 2 mg/kg prednisolone daily for 3 days after upper airway surgery at 10 months of age. Another dog had received immunosuppressive doses of a corticosteroid (2 mg/kg/day) and 2 additional 2 dogs received anti‐inflammatory doses (1 mg/kg) of corticosteroids before developing clinical signs consistent with neosporosis.

### 3.1 Presenting clinical signs

Six dogs (28.6%) presented with clinical signs attributed to generalized myopathy without neurological deficits, with clinical signs present for between 6 and 22 days before referral. Affected dogs were tetraparetic (mild to moderate) with normal spinal reflexes, normal proprioception, exercise intolerance, and had difficulty rising and standing. One dog initially presenting with myopathic signs subsequently developed bilateral decreased menace response, spontaneous horizontal nystagmus, positional ventral strabismus, nystagmus and decreased gag consistent with intracranial disease, with signs progressing over 23 days after initial onset. One dog presented with a 14‐day history of inappetence and reluctance to open its mouth. Two dogs (9.5%) initially presenting with generalized weakness had progressive, non‐ambulatory tetraparesis, with absent tendon (patellar, biceps) reflexes and decreased to absent flexor reflexes in all 4 limbs consistent with polyneuropathy which developed over 4 days in 1 dog and non‐ambulatory paraparesis with absent patellar and withdrawal reflexes in the pelvic limbs which developed over 23 days in the other. The second of these dogs had an increased body temperature (39.9°C) on presentation.

Twelve dogs had clinical signs suggestive of central nervous system (CNS) disease. Of these, 4 (19.0%) had signs consistent with a myelopathy with chronic clinical signs present for 21‐81 days before referral: 1 caudal cervical (C6‐T2) with ambulatory paraparesis and a short forelimb gait, 1 thoraco‐lumbar (T3‐L3) with ambulatory paraparesis and proprioceptive deficits and 2 caudal lumbar (L4‐S1) with ambulatory paraparesis, proprioceptive deficits and decreased patellar reflexes. Seven dogs (33.3%) had neurologic abnormalities consistent with multifocal intracranial disease and 1 dog (4.8%) had clinical signs localized to the cerebellum with generalized cerebellar ataxia, hypermetric gait and right‐sided head tilt. Of 7 dogs with multifocal intra‐cranial signs, signs included proprioceptive deficits (4 dogs), ataxia in all 4 limbs (4 dogs) progressing to non‐ambulatory tetraparesis in 2 dogs, head tilt (1 dog), menace deficit (3 dogs), altered mentation (2 dogs), head turn (1 dog), decreased facial sensation (1 dog), facial hyperesthesia (1 dog), generalized seizure activity (1 dog), and myoclonus (1 dog). Duration of clinical signs before presentation was variable for dogs with intracranial disease, with clinical signs present for between 6 and 86 days prior before referral.

### 3.2 Diagnostic results

Hematology was performed in 18 dogs and was normal in 12 of 16 dogs available for review, and reported as normal by the attending neurologist in the 2 cases unavailable for review. Three dogs had mild neutrophilia with degenerative changes and 1 dog had mild lymphocytosis.

Serum biochemical testing was performed in 20 dogs (Table S2). Of these, 17 had increased ALT activities (median, 360; range, 36‐16 815 U/L; reference interval [RI] 10‐125 U/L). Functional testing was performed in 4 dogs, bile acid testing in 3 and ammonia tolerance testing in 1, and these results were all within normal limits.

Creatine kinase (CK) activity was measured in 14 dogs. It was increased in all dogs tested (median, 3369 U/L; range, 269‐360 154 U/L; RI, <200 U/L). Four of the dogs that had hepatic disease investigation had concurrent measurement of CK activity, which was increased in all (range, 1197‐30 216 U/L). Of 4 dogs with ALT activities within the reference interval, 2 (of 2 tested) had a mildly increased CK activity.

Serological testing for *Neospora caninum* antibodies was performed in all dogs. All dogs had a positive titer for *N. caninum* and 6 dogs had concurrent positive serology for *Toxoplasma gondii*. Twenty‐one dogs had a titer ≥1 : 800 (range, 1 : 800‐1 : 25 600). The positive *T. gondii* titers were 1 : 32 (1 dog), 1 : 128 (3 dogs), 1 : 512 (1 dog), and 1 : 4096 (1 dog).

The 2 M antibody titer testing, performed in 1 dog, was negative.

Abdominal ultra‐sonography was performed in 4 dogs and did not identify any abnormalities.

Cytology of hepatic aspirates indicated evidence of mild mixed inflammation in 2 dogs, cholestasis in 2 dogs and mixed inflammation with protozoal tachyzoites in 1 dog.

Thirteen dogs had advanced imaging. Magnetic resonance imaging of the head was performed in 10 dogs, and of the cervical (C1‐T2), thoracolumbar (T2‐S1), and caudal spinal (L1‐sacrum) regions in 3, 4, and 3 dogs, respectively (Table S3). Of these, 1 dog had no clinically relevant abnormalities identified, whereas 3 dogs had abnormalities consistent with an inflammatory myopathy, without abnormalities of the CNS. Right temporal and masseter musculature involvement was seen in 1 dog presented with reluctance to open the mouth and generalized diffuse muscle involvement (including epaxial muscles and muscles of the thoracic and pelvic limbs) in 2 dogs with clinical signs consistent with progressive polyneuropathy. The remaining 9 dogs had MRI abnormalities of the brain. No evidence of spinal cord MRI abnormalities was found in any dogs. Seven dogs had multifocal abnormalities and 2 had changes affecting only the cerebellum, 1 of which was possibly cerebellar vermis atrophy in a patient presenting with clinical signs of cervical myelopathy. Two dogs with multifocal imaging abnormalities of the brain had evidence of concurrent inflammatory myopathy involving the masticatory muscles.

Analysis of CSF collected by cisternal puncture in 4 dogs and lumbar puncture in 2 dogs had increased protein concentration (range, 0.36‐2.87 g/L; RI, < 0.3 g/L for cisternal samples; 0.6 g/L; RI, < 0.6 g/L for lumbar samples) in 4 dogs in which it was measured, and 4 dogs had a mixed cellular pleocytosis (range, 6.6‐124 × 10^6^/μL; RI, < 6 WBC/μL; Table S3). Four dogs had CSF PCR testing for infectious agents which was positive for *N. caninum* in 1 dog whereas 1 dog tested positive for *T. gondii*.

### 3.3 Treatment

Antimicrobial treatment for neosporosis was prescribed for 20 dogs. All were treated with clindamycin (median dose, 15 mg/kg; range, 10‐18.75 mg/kg PO q12h). Fifteen dogs were treated with trimethoprim‐sulfonamide (median dose, 16 mg/kg; range, 14‐24 mg/kg PO q12h). Dogs with complete response to treatment were treated for 12‐17 weeks, whereas dogs with a partial response typically were treated until at least 2 months after plateau of clinical improvement.

Two dogs received immunosuppressive doses of prednisolone for a presumptive diagnosis of meningoencephalitis of unknown origin (MUO), with 1 of these receiving concurrent clindamycin before *N. caninum* titers were available. One dog was treated for suspected immune‐mediated polymyositis with immunosuppressive doses of prednisolone and azathioprine before referral (14 days).

Half of the dogs treated for neosporosis received an anti‐inflammatory course of prednisolone in addition to anti‐microbial medication (median starting dose, 0.8 mg/kg/day; range, 0.5‐1 mg/kg/day) as a tapering course (median, 3; range, 2‐16 weeks). One dog with suspected myoclonic seizures also was treated with levetiracetam (25 mg/kg PO q8hr) and another dog with generalized seizures was treated with phenobarbitone (4.3 mg/kg PO q12h) and zonisamide (13.3 mg/kg PO q12h).

### 3.4 Outcome

**FIGURE 1:**
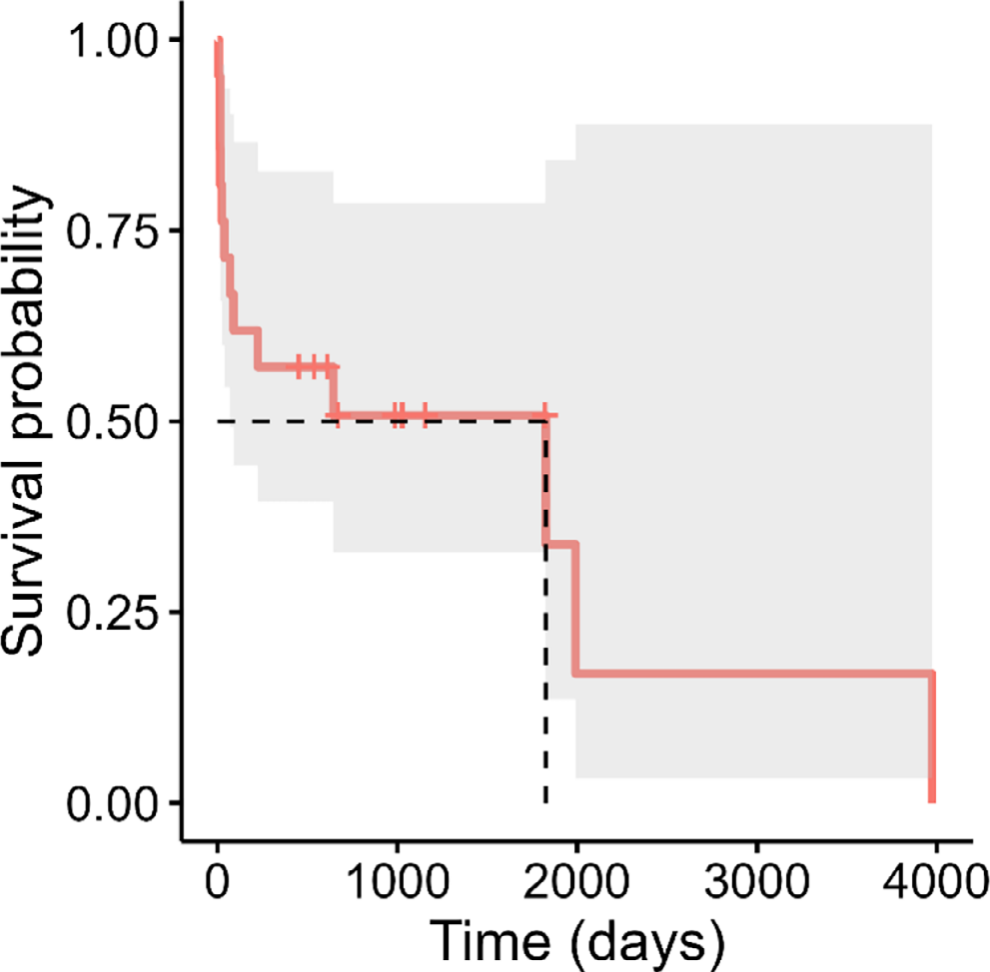
Survival of 21 adult dogs (older than 6 months) with neosporosis (2010‐2023). Dashed line represents median survival time (days), shaded area represents 95% confidence interval for survival times, and tick marks represent censored animals.

All dogs were followed for at least 12 months, with most survivors followed for several years. Neosporosis was the cause of death in the short‐term period (<30 days from onset of signs) in 5 dogs (23.8%) and in the long‐term period (>30 days from onset of clinical signs) in 5 dogs (23.8%), with an overall mortality rate of 47.6%. Survival at 3 (14/21, 63.6%) and 6 months (13/21, 59%) was similar to 12‐month survival with 57% (12/21 dogs) alive after 1 year. Median survival time was 1825 days (Figure 1).

Seven dogs showed minimal or no response to treatment and died or were euthanized at a median 15 days from the onset of clinical signs (range, 7‐70 days). Two dogs treated with immunosuppressive doses of corticosteroids for presumed MUO failed to improve and were euthanized before serological testing results became available. One dog (D20) that received immunosuppressive treatment for suspected immune‐mediated polymyositis and developed progressive CNS signs before serological testing was treated with clindamycin but remained on immunosuppressive doses of corticosteroids and was euthanized 2 days later. One dog (D8) experienced acute cardiorespiratory arrest 3 days after starting treatment for neosporosis. Despite initially improved enzyme activities (ALT, CK), this dog developed progressive neuromuscular abnormalities. Necropsy was not performed, but muscle biopsy samples were collected for histopathology post‐mortem and were consistent with protozoal myositis as reviewed by a board‐certified pathologist. Dog D2 with focal myopathy of the masticatory muscles showed minimal response and died at home 24 days after starting treatment. Dogs D17 and D21 demonstrated mild clinical improvement but were euthanized after 7 and 13 days of treatment, respectively, because of persistently decreased mobility and intracranial signs in dog D21.

Survival information was available for 12 months or longer for all dogs that showed clinical response to treatment. Eight dogs (38%) showed complete resolution of clinical signs with treatment within 2 to 4 weeks with normalization of ALT and CK activities by week 4 in the 7 dogs reassessed. Of these dogs that showed complete resolution, 5 had clinical signs of myopathy, 2 multifocal intracranial signs, and 1 had both myopathic and intracranial signs. Four of the 5 dogs that received corticosteroid treatment for another condition before the onset of clinical signs of neosporosis had a complete response to treatment.

Six dogs (30%) showed clinical improvement with treatment but had persistent neurological deficits. Two dogs had permanent paraparesis of which 1 (D13) was managed using a mobility cart for 5 years whereas the second was euthanized after 9 weeks of treatment because of lack of improvement. One dog (D5) that showed mild clinical improvement initially, experienced declining mobility and loss of muscle mass and was euthanized 10 months after diagnosis. This dog was treated with clindamycin until euthanasia. Dog D10 showed marked clinical improvement but had persistent mild cerebellar ataxia that was stable for 12 months after discontinuation of treatment before being lost to follow‐up. Dogs D4 and D14 had persistent proprioceptive deficits.

Relapse was common. Nine relapse events occurred in 4 dogs, in 2 dogs with complete and in 2 with incomplete resolution of clinical signs. Relapses occurred after discontinuation of treatment at a median of 8 months (range, 2‐18 months) after discontinuation. All 4 dogs relapsed with the same clinical signs as their initial presentation, but clinical signs typically were less severe than at the time of initial diagnosis. Repeat diagnostic tests were not commonly performed. Two of 4 dogs had repeat serum biochemistry, which identified increased CK activity in both (range, 506‐1486 U/L; RI, < 200 U/L). Among 9 relapses, 3 dogs were treated and improved back to their initial response (1 complete and 2 incomplete) on multiple occasions, whereas 1 dog with an initial complete response failed to respond to retreatment and was euthanized. Multiple relapses occurred over 2 years in 2 dogs (D4, D14) and over 8 years in a third dog (D16). A dog (D3) with myoclonus that had a complete response to treatment that included levetiracetam, developed mild myoclonus when levetiracetam was discontinued despite resolution of other neurological abnormalities. This dog had a single generalized seizure 15 months after diagnosis despite a normal neurological examination on representation. Repeat MRI disclosed persistent marked T2W hyperintensity and T1W hypointensity in the cerebellum, but previous hyperintensities in the cerebral hemispheres had resolved. Repeat CSF analysis was normal and repeat serology showed a decrease in titer from 1 : 6400 to 1 : 50. No further generalized seizures were reported in the follow‐up period of the study. An acquired seizure disorder secondary to previous neosporosis was considered the most likely cause in this dog.

## 4 DISCUSSION

Dogs with adult‐onset neosporosis presented with a wide range of clinical signs indicating focal or generalized myopathy, polyneuropathy, myelopathy, intracranial disease or as a multifocal disease process. Variable imaging and CSF abnormalities were seen. Despite response to initial treatment, relapse was seen in several cases. Serum activities of ALT (85% of tested) and CK (100% of tested) frequently were increased in this cohort of dogs with myopathic or neurologic abnormalities or both and may be useful screening tests for neosporosis and monitoring tools for potential relapse. A recent case series describing 41 affected dogs in the United Kingdom, including dogs with juvenile onset of clinical signs, also reported a wide variety of clinical presentations, and a high frequency of relapse, but described a much lower response to treatment.^10^

Protozoal myositis may be more prevalent than previously recognized, with one‐third of cases in our series presenting with clinical evidence of myopathy. Typically, the juvenile form of neosporosis manifests as polyradiculoneuritis and myositis, whereas the adult‐onset form is associated with encephalitis.^3^ In a review of 200 cases of inflammatory myopathies in dogs, 24.4% of dogs tested for *N. caninum* had positive serology, but *N. caninum* cysts were detected in muscle biopsy specimens of only 2 dogs.^11^ It was suggested that either positive serology in these dogs represented exposure only (titers ranged from 1 : 64 to 1 : 3200), or biopsy samples are insensitive for the detection of infectious organisms.^11^ Neosporosis should be considered in dogs presented for weakness or other signs of myopathy.

The MRI findings were inconsistent, with only 1 dog (D11) showing the cerebellar atrophy previously described as associated with neosporosis in adult dogs.^12^, ^13^, ^14^ In previous case series of adult‐onset neosporosis, 10 of 19 dogs had cerebellar atrophy, 6 dogs had focal abnormalities in the brain or spinal cord, 7 dogs had multifocal brain abnormalities, 3 had multifocal abnormalities throughout the brain and spinal cord, 3 had abnormalities of the musculature and 1 dog's MRI was normal.^6^, ^12^, ^13^, ^14^, ^15^ Cerebellar atrophy may be a less common abnormality in dogs with adult‐onset neosporosis than suggested in these previous imaging case series.^12^, ^13^, ^14^ The imaging findings seen in our cohort of dogs with neosporosis are not specific for this disease and are frequent in other more common causes of encephalitis (eg, MUO) and other diseases.

Protozoal encephalitis is an important differential diagnosis for MUO, as indicated by the 2 dogs treated for presumptive MUO pending protozoal titers that showed progressive neurological signs and were subsequently euthanized after immunosuppressive treatment. Neither dog had a serum CK activity measured. A previous study reported low seroprevalence of neosporosis in a cohort of MUO‐diagnosed dogs and suggested that initiating immunosuppressive treatment before protozoal titers were available posed minimal risk.^16^ Experience in our cohort of dogs likely reflects geographical variations in disease prevalence.^5^, ^8^ Although histopathological confirmation of neosporosis was lacking, positive serology and poor response to treatment for MUO was supportive of a diagnosis of neosporosis. A recent study found serum CK activities were significantly increased in dogs with meningoencephalitis and positive serology for Neospora (≥1 : 800) compared with non‐infectious cases.^9^ A CK activity of 485 U/L had a 95.24% sensitivity and 96.61% specificity for predicting a positive IFAT ≥1 : 800.^9^ In our cohort of dogs with signs and imaging findings consistent with encephalitis, all dogs tested had increased serum CK. Given the ease of CK activity measurement, it may serve as a useful screening tool in suspected MUO cases to guide decisions on the safety of immunosuppressive treatment, along with consideration of concurrent antiprotozoal treatment while awaiting infectious disease test results.

Cerebrospinal fluid Multiplex PCR for infectious agents was inconsistently performed in our cases and had limited diagnostic utility in the dogs in which it was performed. Among the 4 tested dogs, only 1 was positive for *N. caninum* and 1 had a positive PCR result for *T. gondii* despite negative serology. Three patients with negative PCR, multifocal MRI abnormalities, inflammatory CSF, and positive serology results, responded positively to treatment for neosporosis. Previous studies have reported variable CSF PCR results in small numbers of dogs with presumed clinical neosporosis.^4^, ^12^, ^13^, ^16^, ^17^ Given the unknown sensitivity and specificity of this test, its diagnostic utility is unknown and a diagnosis of adult‐onset neosporosis should not be excluded on the basis of negative CSF PCR. Measurement of CK activity may be a more useful screening tool for dogs with meningoencephalitis, based on ease of collection, cost, and sensitivity, compared with Multiplex PCR while awaiting other infectious disease results.

Myocarditis previously has been described in adult‐onset neosporosis and is associated with sudden death.^18^, ^19^, ^20^ One dog with generalized myopathy died suddenly and myocarditis was suspected, but examination of cardiac muscle was not performed.

Only 1 dog using the initial search criteria presented with cutaneous disease, suggesting low incidence at our referral institution despite a substantial dermatology case load. This finding contrasts with previous reports where cutaneous involvement was noted in 14/57 (25%) of cases.^21^ The higher prevalence of cutaneous disease in the veterinary literature may stem from the comparative ease and reliability of skin biopsy for diagnosis, in contrast to muscle or CNS biopsy.

The ideal treatment and treatment duration for neosporosis remains undefined, with some sources recommending treatment for as long as the dog is improving.^3^ Others have documented complete clinical resolution with treatment duration ≤4 weeks, but these individual adult cases did not have long term follow‐up beyond the initial treatment period.^21^, ^22^ Dogs in our study were typically treated for longer than previously has been described, in response to known cases of relapsed disease. It is possible that some of our dogs may have had complete clinical response with shorter treatment courses. Further research is needed to determine the ideal treatment duration and assess potential benefits of multimodal antimicrobial therapy.

Nearly one‐third of dogs in our study experienced relapses despite initially showing complete or functional response to treatment, indicating a guarded prognosis for adult‐onset neosporosis. Dogs with myopathy only or those that developed disease while receiving corticosteroids could have a better prognosis, because a higher proportion of these dogs in our case series showed long term resolution of clinical signs. Two dogs in our study had received anti‐inflammatory doses of corticosteroids before development of clinical signs, a factor not previously reported as a potential risk for protozoal infection, and corticosteroids also commonly have been used at an anti‐inflammatory dose in other affected dogs early in treatment of disease.^23^, ^24^, ^25^ Further research and larger case numbers are necessary to evaluate the impact of prior corticosteroid use on disease manifestations and prognosis.

The cause of infection remained undetermined in most cases, with horizontal transmission considered unlikely in most cases. It is more likely dogs developed clinical signs because of activation of encysted bradyzoites, which likely was impacted by immunosuppression in 3 dogs. Considering the 14% seroprevalence of neosporosis among apparently healthy dogs in Sydney, determining serological status and monitoring for disease development is an important consideration in any dog undergoing immunosuppressive treatment in Australia and warrants consideration in regions with known seroprevalence.^5^

Previous studies have suggested a possible breed predisposition in Labradors retrievers and boxers for adult‐onset neosporosis, which was not observed in our cohort of dogs.^12^, ^13^, ^21^ Greyhounds also previously have been suggested as a more susceptible breed and were the most commonly represented breed in our study population (4/21 dogs).^26^ This finding may be related to the small sample size, with a recent study not finding a significant difference in seroprevalence of *N. caninum* between retired racing greyhounds and other healthy dogs in the same geographic area.^5^

Six dogs had concurrent positive serum titers for *T. gondii*. Clinical toxoplasmosis in dogs typically is associated with immunosuppression with 8/11 published confirmed cases of toxoplasmosis by tissue biopsy and IHC in dogs having received immunosuppressive treatment.^27^ Positive toxoplasma titers were thought to reflect previous exposure to *T. gondii* and were of uncertain clinical relevance, with no current evidence for cross‐reactivity on IFAT testing in dogs.^27^

### 4.1 Limitations

A limitation of our retrospective study, common to many clinical studies, was the inconsistent availability of a histopathologic diagnosis, which is particularly challenging in animals with encephalitis or myelitis. Diagnosis of neosporosis was based primarily on clinical signs, serology, response to antimicrobial treatment and exclusion of other causes.^2^, ^3^ Serum antibody titer quantification does not distinguish between exposure and active disease, but titers >1 : 200 have been considered diagnostic in patients with compatible clinical signs given the difficulty in identifying the organism in tissue ante‐mortem.^3^, ^11^, ^28^ A recent study found the seroprevalence to *N. caninum* in Sydney to be 14%, which is similar to results in 1997, but this study reported greater variation in titers when compared to the prior seroprevalence data with titers up to 1 : 3200 detected in healthy dogs.^5^, ^8^ Consequently, it is possible that some of the dogs in our study, particularly those that did not respond to treatment, may not have had neosporosis. However no alternative diagnosis was determined in these dogs. Other infectious causes of encephalitis are rare in Australia, with no evidence for viral or tick‐borne encephalitis and antigen testing available for Cryptococcus spp, which is endemic, with the highly sensitive lateral flow antigen assay.^29^ Ideally, necropsy would have been performed to confirm the suspected diagnosis in affected dogs that died or were euthanized.

## 5 CONCLUSION

Adult‐onset neosporosis, although uncommon, should be considered in dogs presenting with neurological abnormalities or clinical signs suggestive of myopathy and unexplained increases in ALT activity. Serum CK activity was increased in all dogs tested and may be a useful screening diagnostic test. Treatment duration and response for neosporosis was variable with frequent relapses, even among dogs that responded initially to treatment, and the overall prognosis appears guarded. Dogs presenting solely with clinical myopathy or recent corticosteroid use may have a more favorable prognosis.

## CONFLICT OF INTEREST DECLARATION

Authors declare no conflict of interest.

## OFF‐LABEL ANTIMICROBIAL DECLARATION

Animals were treated with off‐label antimicrobials clindamycin, trimethroprim‐sulphonamide and pyrimethamine.

## INSTITUTIONAL ANIMAL CARE AND USE COMMITTEE (IACUC) OR OTHER APPROVAL DECLARATION

Authors declare no IACUC or other approval was needed. Owner information and consent forms including permission for sharing de‐identified clinical information are part of standard permission forms which are signed before hospital admission.

## HUMAN ETHICS APPROVAL DECLARATION

Authors declare human ethics approval was not needed for this study.

## Supporting information


**Table S1:** Clinical presentation of dogs presenting with adult onset neosporosis.
**Table S2:** Laboratory results of dogs with adult onset neosporosis.
**Table S3:** Advanced imaging findings and CSF results of dogs with adult onset neosporosis.
**Table S4:** Survival and outcome of dogs with adult onset neosporosis.

## Endnote

^1^
Eleven performed on 1.5 T and 2 on 0.25 T scanner across three facilities.
