# Supplementary material for: Neosporosis in 21 adult dogs, 2010‐2023
Source: J Vet Intern Med. 2024 Oct 23;38(6):3079–86. doi: 10.1111/jvim.17219 (PMC11586566; doi:10.1111/jvim.17219)
Supplement: Supplementary file 1 — Table S1: Clinical presentation of dogs presenting with adult onset neosporosis. Table S2: Laboratory results of dogs with adult onset neosporosis. Table S3: Advanced imaging findings and CSF results of dogs with adult onset neosporosis. Table S4: Survival and outcome of dogs with adult onset neosporosis. [file JVIM-38-3079-s001.docx]

| **Dog** | **Signalment** | **Duration of signs** | **Presenting Signs** | **Neurolocalisation** |
| --- | --- | --- | --- | --- |
| 1 | 1yo FS bullmastiff | 3 weeks | myopathy, joint pain - stiff gait, difficult to rise and lie down | Myopathy |
| 2 | 10 yo FS pug | 2 weeks | subtle behaviour changes - not eating | Myopathy |
| 3 | 5yo FS CKCS | 5 weeks | cervical myelopathy, head/neck myoclonos | Multifocal |
| 4 | 9mo ME Hungarian vizsla | 10 days | CP deficits Right side, subtle head tilt to the right , long stride gait, mild hind limb ataxia, muscle atrophy temporalis right side | Multifocal |
| 5 | 6yo MN greyhound | 2 months | L4 caudal myelopathy, reduced HL muscle mass, reduced patellar reflexes | L4 caudal myelopathy |
| 6 | 3yo MN CKCS x poodle | 2 weeks | reluctance to walk, lethargic, dull, poor appetite | Myopathy |
| 7 | 2yo MN Bernese Mountain Dog | 1 week | reluctance to walk, weak | Myopathy |
| 8 | 10mo MN French Bulldog | 4 days | Progressive flaccid paralysis | LMN |
| 9 | 10mo MN French bulldog | 1 week | Lethargy | Myopathy |
| 10 | 10yo FS cocker spaniel x poodle | 2 weeks | Generalised marked cerebellar ataxia, hypermetric LF, LH, mild R head tilt | Cerebellum |
| 11 | 1yo MN pug | 3 weeks | paraparesis, brisk forelimb reflexes, RFL paresis, CP deficit RFL | cervical myelopathy |
| 12 | 5yo FS CKCS | 2 weeks | Non-ambulatory vestibular tetra-ataxia, absent menace OS, hyperaesthetic facial sensation | Multifocal |
| 13 | 8mo MN Labrador x poodle | 3 weeks | pyrexia (39.4), weakness --> progressive flaccid tetraparesis | LMN |
| 14 | 1yo ME Doberman | 6 weeks | pelvic limb ataxia and paresis with CP deficits | T3-L3 myelopathy |
| 15 | 1.5yo FS WHWT | 1 week | multifocal - mildly altered (quiet mentation), mild tetra ataxic + hypermetric forelimbs , inconsistent menace, proprioceptive deficits pelvic limbs | Multifocal |
| 16 | 4yo FS WHWT | 12 weeks | abnormal gait - weakness, poor proprioception and loss of balance | multifocal |
| 17 | 4yo FS greyhound | 12 weeks | pelvic limb ataxia to paresis, caudal to L3 myelopathy | L3 Caudal myelopathy |
| 18 | 4yo MN greyhound | 3 days | Non-ambulatory tetra-ataxia and weakness, seizures | Multifocal CNS; myopathy |
| 19 | 1yo MN kelpie x | 3 weeks | generalised weakness after steroids, muscle wastage | Generalised (myopathy, liver) |
| 20 | 7yo MN Labrador | 3 week | diffuse neuromuscular weakness, menace reduce, spontaneous horizontal nystagmus, positional ventral strabismus & nystagmus, reduced gag | Multifocal CNS; myopathy |
| 21 | 9yo FS greyhound | 1 week | central vestibular / brainstem, progressive tetraparesis | Multifocal CNS |

Table 1. Clinical presentation of dogs presenting with adult onset neosporosis

| **Dog** | **Globulins**  **RR 25-45 g/L** | **ALT**  **RR 10-125 U/L** | **CK**  **RR 10-200 U/L** | **Neospora Titer** | **Toxoplasma titer** |
| --- | --- | --- | --- | --- | --- |
| 1 | 33 | 36 | 269 | 1/800 | Negative |
| 2 | 37 | 247 | 729 | 1/1600 | 1/4096 |
| 3 | 38 | 280 | 2420 | 1/6400 | Negative |
| 4 | 37 | 440 | 3369 | 1/3200 | Negative |
| 5 | 31 | 560 | 7420 | 1/6400 | Negative |
| 6 | 36 | 1825 | 9210 | 1/12800 | Negative |
| 7 | 35 | 957 | 11622 | 1/25600 | Negative |
| 8 | 30 | 16815 | 30126 | 1/800 | Negative |
| 9 | 31 | 1427 | 30498 | 1/3200 | Negative |
| 10 | 48 | 196 | n/a | 1/12800 | N/a |
| 11 | n/a | 152 | n/a | 1/25000 | 1/128 |
| 12 | 35 | 99 | n/a | 1/3200 | 1/128 |
| 13 | n/a | 483 | n/a | 1/3200 | Negative |
| 14 | n/a | n/a | n/a | 1/3200 | Negative |
| 15 | 52 | 256 | n/a | 1/6400 | N/a |
|  |  |  |  |  |  |
| 16 | 40 | 73 | 422 | 1/6400 | Negative |
| 17 | n/a | 187 | n/a | 1/12800 | 1/128 |
| 18 | 33 | 1264 | 360,154 | 1/12800 | Negative |
| 19 | 41 | 3616 | 1197 | 1/25000 | 1/512 |
| 20 | 35 | 242 | 2005 | 1/6400 | 1/32 |
| 21 | 35 | 783; TBil 18 | 1112 | 1/6400 | Negative |

Table 2. Laboratory results of dogs with adult onset neosporosis

| **Dog** | **MRI region scanned** | **Summary of MRI findings** | **CSF Results** |
| --- | --- | --- | --- |
| 2 | Brain, cervical spine | Patchy increased T2W FSE & STIR hyperintensity of the right temporal and masseter musculature with contrast enhancement. No definite brain abnormalities. | N/a |
| 3 | Brain, cervical spine | Initially: Severe T2W hyperintensity at the periphery of the cerebellum & multiple T2W hyperintensities within the cerebrum with varying degrees of contrast uptake, marked in the cerebellum.  5 months later: Cerebellar changes similar to previous scan. No abnormalities in the cerebral hemisphere or brainstem. | Lumbar; protein 0.6g/L (0.0-0.5g/L), mild RBC contamination, TNCC 0; PCR negative |
| 4 | Brain | Marked T2W hyperintensity of the white matter of the left cerebral hemisphere with midline shift to the right and areas of marked contrast enhancement. Marked muscle atrophy of the R masticatory musculature with patchy marked contrast enhancement. | Cisternal; protein 2.17g/L (RR 0.0-0.3g/L); moderate to marked mixed pleocytosis with eosinophils; TNCC 124x 10^6/L, RCC 18x10^6/L; PCR negative N.caninum, positive T.gondii |
| 8 | Thoracic spine, lumbar spine | Hypointensities in the epaxial muscles, muscle of the thoracic and pelvic extremities have T2W hyperintense striated appearance which is strongly contrast enhancing. | N/a |
| 10 | Brain | Marked increased T2W signal of the meninges of the cerebellum, highlighting the cerebellar gyri and increased sulcal width; Focal area (5mm x 2mm) of increased T2W signal intensity of the left brainstem, just caudal to the cerebellar peduncle. | N/a |
| 11 | Brain, Cervical spine, screening sagittal of thoracic and lumbar spine | Possible atrophy in the cerebellar vermis and relatively small cerebellar hemispheres with increased T2W signal of meninges. | N/a |
| 12 | Brain | Patchy T2W hyperintensity within the brainstem and within the cerebellum, strongly contrast enhancing.  Increased contrast uptake of the left optic nerve. T2W hyperintensity at the rostral horn of the left ventricle. T2W hyperintensity within the cervical spine without contrast enhancement. Patchy T2W hyperintensities within the masticatory muscles with some contrast enhancement. | N/a |
| 13 | Thoracic spine, lumbar spine | On STIR sequences, there is patchy to linear hyperintensity throughout the epaxial musculature, hypoaxial or iliopsoas muscles, gluteal muscles, muscles of the proximal hindlimbs, muscles of the scapulae bilaterally with streaky contrast enhancement. | N/a |
| 15 | Brain | There is moderate dilatation of the lateral ventricles, with slight asymmetry (left larger than right).  On T2W images, there is marked, asymmetric, increased signal of the peripheral grey matter of the cerebrum with all lobes affected. Thalamus also affected. Marked T2W hyperintensity of the surface of the cerebellum and thinning of the cerebellar folia. | Cisternal; insufficient sample for protein; moderate mixed cell pleocytosis with predominately monocytic cells; TNCC 41x10^6/L, RCC 9x10^6/L |
| 16 | Brain | Abnormal signal intensity involving the superficial portion of the cerebellar structures involving both the hemispheres and vermis. There is enhancement over the surface of the cerebellum and ventrally over the pons and brainstem. | N/a |
| 17 | Caudal thoracic, lumbar, sacrum | No definite abnormalities found. | Lumbar; consistent with blood contamination |
| 18 | Brain | Multifocal T2 and Flair hypointense lesions (worse on left) throughout olfactory, frontal, temporal lobes of the forebrain and mid brain, mild ventriculomegaly and cortical atrophy. | Cisternal; protein 0.36g/L (0.0-0.3g/L); mild mixed pleocytosis with predominately macrophages; TNCC 6.6 x 10^6/L, RCC 53x10^6/L; PCR negative |
| 21 | Brain | Mild T2W and FLAIR hyperintensity within the brain stem, scant contrast within the cerebellum | cisternal: Mild to moderate mixed cell pleocytosis with predominately monocytic cell; TNCC 80 x 10^6/L, RCC 1000 x 10^6/L; TP 2.87g/L; PCR positive |
|  |  |  |  |

Table 3. Advanced imaging findings and CSF results of dogs with adult onset neosporosis

| **Dog** | **Survival time (days)** | **Outcome** | **Relapse** |
| --- | --- | --- | --- |
| 1 | 1820 - censored | Complete resolution | No |
| 2 | 38 | Died | n/a |
| 3 | 1028 - censored | Complete resolution | no |
| 4 | 1154 - censored | Incomplete resolution | Yes |
| 5 | 224 | Incomplete resolution - euthanised | n/a |
| 6 | 987 - censored | Complete resolution | no |
| 7 | 1991 | Complete resolution; died secondary to neoplasia | no |
| 8 | 7 | No response - died | n/a |
| 9 | 610 - censored | Complete resolution | No |
| 10 | 453 - censored | Incomplete resolution | no |
| 11 | 70 | Incomplete resolution - euthanised | n/a |
| 12 | 15 | No response - Euthanised | n/a |
| 13 | 1825 | Incomplete resolution; died secondary to neoplasia | no |
| 14 | 671 - censored | Incomplete resolution | yes |
| 15 | 13 | No response - euthanised | n/a |
| 16 | 3973 | Complete resolution; died secondary to neoplasia | yes |
| 17 | 89 | Incomplete resolution - euthanised | n/a |
| 18 | 644 | Complete resolution; relapse and poor response - euthanised | yes |
| 19 | 537 - censored | Complete resolution | no |
| 20 | 24 | No response - euthanised | n/a |
| 21 | 13 | No/minimal response - euthanised | n/a |
|  |  |  |  |

Table 4. Survival and outcome of dogs with adult onset neosporosis
